# Antimicrobial Peptides: From Design to Clinical Application

**DOI:** 10.3390/antibiotics11030349

**Published:** 2022-03-06

**Authors:** Chunye Zhang, Ming Yang

**Affiliations:** 1 Department of Veterinary Pathobiology, University of Missouri, Columbia, MO 65212, USA; zhangcherryuniversity@gmail.com; 2 Department of Surgery, University of Missouri, Columbia, MO 65211, USA

**Keywords:** antibiotic resistance, antimicrobial peptides, design, optimization, delivery, clinical application

## Abstract

Infection of multidrug-resistant (MDR) bacteria, such as methicillin-resistant *Staphylococcus aureus* (MRSA), carbapenem-resistant *Enterobacteriaceae* (CRE), and extended-spectrum beta-lactamase (ESBL)-producing *Escherichia coli*, brings public health issues and causes economic burden. Pathogenic bacteria develop several methods to resist antibiotic killing or inhibition, such as mutation of antibiotic function sites, activation of drug efflux pumps, and enzyme-mediated drug degradation. Antibiotic resistance components can be transferred between bacteria by mobile genetic elements including plasmids, transposons, and integrons, as well as bacteriophages. The development of antibiotic resistance limits the treatment options for bacterial infection, especially for MDR bacteria. Therefore, novel or alternative antibacterial agents are urgently needed. Antimicrobial peptides (AMPs) display multiple killing mechanisms against bacterial infections, including directly bactericidal activity and immunomodulatory function, as potential alternatives to antibiotics. In this review, the development of antibiotic resistance, the killing mechanisms of AMPs, and especially, the design, optimization, and delivery of AMPs are reviewed. Strategies such as structural change, amino acid substitution, conjugation with cell-penetration peptide, terminal acetylation and amidation, and encapsulation with nanoparticles will improve the antimicrobial efficacy, reduce toxicity, and accomplish local delivery of AMPs. In addition, clinical trials in AMP studies or applications of AMPs within the last five years were summarized. Overall, AMPs display diverse mechanisms of action against infection of pathogenic bacteria, and future research studies and clinical investigations will accelerate AMP application.

## 1. Introduction

According to the 2019 antibiotic resistance report by the Center for Disease Control and Prevention (CDC), more than 2.8 million cases of antibiotic-resistant infection occur in the United States, with 35,000 infection-caused deaths [1]. The prevalence of antibiotic-resistant bacterial infections poses a big threat to animal and human health and causes economic loss [2,3]. Especially, infection of multidrug-resistant (MDR) bacteria, such as methicillin-resistant *Staphylococcus aureus* (MRSA), vancomycin-resistant *Enterococcus*, carbapenem-resistant *Enterobacteriaceae* (CRE), and MDR *Pseudomonas aeruginosa*, is a global issue [4,5,6,7].

Inappropriate use and overdosage of antibiotics drive and accelerate antibiotic resistance [8,9]. For example, antibiotics are prescribed for viral infections, which may not be necessary. Data analysis from ten public health facilities showed that a high proportion (36.66%) of prescriptions for the treatment of upper respiratory tract infection included at least an antibiotic [10]. In addition, more than 50% of antibiotics were applied to treat cough and 20% of antibiotics were prescribed for pharyngitis. A meta-analysis study showed that initial inappropriate antibiotic therapy in hospitalized patients with Gram-negative bacterial infections can cause adverse outcomes including mortality, with an unadjusted summary odds ratio [OR] 2.66 and 95% confidence interval [CI] 2.12–3.35 [11]. Therefore, monitoring antibiotic use is critically important to reduce the development of antibiotic resistance in bacteria.

A recent report showed that the COVID-19 pandemic caused a spread of MDR bacterial infections including MRSA, carbapenem-resistant *Acinetobacter baumannii*, and fungi *Candida auris* [12]. Overuse of antibiotics is a hidden threat in the pandemic of viral infection. For example, a review report with the analysis of 10 African countries showed that antibiotics such as amoxicillin and ampicillin were commonly prescribed antibiotics for patients with severe acute respiratory syndrome coronavirus 2 (SARS-CoV-2) infection [13]. An outbreak of ESBL-producing *Klebsiella pneumoniae* in COVID-19 infected patients was shown in intensive care units [14]. In contrast, Gaspari et al. reported that during the COVID-19 pandemic period the infection of extended-spectrum beta-lactamase (ESBL)-producing *Escherichia coli* was dramatically reduced compared to that in pre-pandemic times [15] due to the behavioral change (e.g, using hand sanitizer).

Antimicrobial peptides (AMPs) are expressed by most living organisms and play important roles in defending against bacterial, viral and fungal infections [16,17,18], as well as adaptive immunity during the development of cancers and autoimmune diseases [19,20]. AMPs with diverse modes of action distinct from conventional antibiotics exhibit potential capacity against infection of MDR bacteria and other pathogens [21,22]. With the advance of nanotechnology, AMP-derived nanomedicines can be designed to treat bacterial infection locally [23,24].

In this review, the killing mechanisms of antibiotics and resistant mechanisms in bacteria are reviewed, followed by discussion of the spread of antibiotic resistance among bacteria. Then, the function, design, optimization, and delivery of AMPs are summarized. Finally, some clinical trials for the past five years and applications of AMPs are reviewed.

## 2. Antibiotic Action and Resistance

Mechanisms of action of antibiotics consist of inhibition of cell wall synthesis (e.g., beta-lactam antibiotics such as penicillin and carbapenem), protein synthesis (e.g., macrolides and tetracyclines), or nucleic acid synthesis (e.g., quinolones), and damage to cell membrane (e.g., polymyxins) [25]. However, bacteria develop ways to inhibit antibiotic function, including (1) inactivation of antibiotics by enzymes. For example, Gram-negative bacteria *E. coli* and *K. pneumoniae* produce β-lactamases to destroy β-lactam antibiotics [26]. Erythromycin esterases (Eres) such as EreA and EreC can cleave macrocyclic lactone to develop resistance to macrolides [27,28]. (2) Reduction of antibiotic intracellular concentration. For example, overexpression of resistance-nodulation-division (RND) efflux pumps in *Pseudomonas aeruginosa* is responsible for MDR to antibiotics, such as ticarcillin and ciprofloxacin [29]. *Streptococcus pneumoniae* develops resistance to macrolides via antibiotic exclusion by efflux pumps and ribosomal demethylation by erythromycin ribosomal methylase B (*ermB*) gene-encoded enzyme, and less commonly, mutations of the ribosomal macrolide targeting site [30]. (3) Mutation of antibiotic function sites. For example, quinolones (e.g., ciprofloxacin and levofloxacin) can function on DNA gyrase and Topoisomerase IV to inhibit the synthesis of nucleic acids of both Gram-negative (e.g., *K. pneumoniae*) and Gram-positive bacteria (e.g., *Clostridium perfringens*) [31]. However, mutations in genes encoding DNA gyrase and Topoisomerase IV can abolish the binding of quinolones to cause their function loss [32]. (4) Bypass the target site of the antibiotics. For example, structural modification of dihydrofolate reductase (DHFR) or dihydropteroic acid synthase (DHPS) is a mechanism to develop resistance to trimethoprim-sulfamethoxazole (TMP-SMX) in pathogenic bacteria [33]. In addition, *Burkholderia pseudomallei* (*bpe*)*E*, *bpeF* and outer membrane porin C (*oprC)* genes of efflux pumps mediate resistance to TMP-SMX [33]. The modes of action of antibiotics, targeting bacteria, and bacterial resistance mechanisms are summarized in Table 1.

## 3. How Bacteria Acquire Antibiotic Resistance Genes

There are several ways that bacteria can acquire antibiotic resistance genes. Transduction (DNA transfer mediated by phages), conjugation (DNA transfer between bacteria mediated by plasmids), and transformation (released pieces of DNAs from donor cells directly taken up by recipient cells) are three strategies or mechanisms of horizontal gene transfer among bacteria [35,36]. Antibiotic resistance genes can be horizontally transferred through mobile genetic elements such as plasmids [37,38], transposons(Tns) [39], and integrons [40], as well as bacteriophages [41]. For example, *E. coli*, *K. pneumoniae*, and *A. baumannii* both in animals and humans carry plasmids that encode tigecycline resistance genes *tet(X3)* and *tet(X4)* [42,43]. The bacterial Tn family belongs to DNA Tns, which can transfer between plasmids or between DNA chromosome and plasmid [44]. For example, Tn7-like transposons such as Tn6813, Tn6814, and Tn6765 were found in *Enterobacterales* isolated from food animals, which were associated with resistance to sulfamethoxazole and streptomycin [45]. The presence of class 1 integrons in commensal *E. coli* strains is associated with tetracycline-resistant genes *tet(A)* and *tet(B)* [46].

Furthermore, bacteriophages play a pivotal role in the spread of antibiotic resistance genes in pathogenic bacteria via phage-mediated transduction [47]. In addition, they function as environmental reservoirs of antibiotic resistance genes, which pose a large threat to public health [48,49]. For example, antibiotic resistance genes such as *bla*_TEM_ (β-lactam antibiotic resistance gene, such as penicillin), *qnrA* (quinolone), *mecA* (methicillin resistance gene), and *sul1* (sulfonamide resistance gene) were found in phage DNAs in meat [50]. Furthermore, bacteriophages that carry resistance genes can be found in animal feces, water, soil, and vegetables (e.g., cucumber and spinach) [51,52,53,54]. Phage-carried antimicrobial resistance genes *OXA-23* encoding carbapenemase and New Delhi metal-lo-beta-lactamase 1 (*NDM-1*) cause antibiotic resistance in bacteria, such as *A. baumannii* [55]. Meanwhile, several different mobile elements with antibiotic resistance genes are found in the same bacterial strain (e.g., *E. coli*) [56]. Examples of transfer of resistance genes in bacteria via mobile genetic elements are listed in a table (Table 2). The whole-genome sequencing (WGS) is a valuable tool that can be applied to analyze the bacterial genomes to search the DNA fragments that are associated with antibiotic resistance [57,58].

Currently, the clustered regularly interspaced short palindromic repeat (CRISPR) and its associated protein 9 (Cas9) system with a single guide RNA (sgRNA) is broadly applied to investigate the role of specific genes such as DNA gyrase subunit A (*gyrA*) and mobilized colistin resistance gene (*mcr-1*) in antibiotic resistance for quinolone and colistin, respectively [59,60]. In addition, a high-throughput chromatin conformation capture method has been applied to reconstruct each genome in the mixed microbial sample [61] and to study the process of horizontal gene transfer in human microbiome [62].

## 4. Alternative Antibiotics: Antimicrobial Peptides

AMPs play important roles in both innate and adaptive immunity. Natural AMPs are found in plants, vertebrates, invertebrates, and small organisms such as bacteria and fungi. The antimicrobial peptide database (APD)3 (https://aps.unmc.edu/, accessed on 18 December 2021) shows that a total of 3283 AMPs are from six life kingdoms, including 361 from plants and 2431 from animals [63]. The mechanisms of AMPs can be classified into two major types: (1) direct killing via disrupting membrane integrity or impacting the synthesis of intracellular components including both nucleic acids and proteins, and (2) modulating immunity to clear pathogenic infection [19,64]. In addition, AMPs display multiple other functions such as membrane depolarization and destabilization as discussed in the following sections. The diverse functions of AMPs cause bacteria to develop hardly any resistance to them.

### 4.1. Structures of AMPs

**Figure 1:**
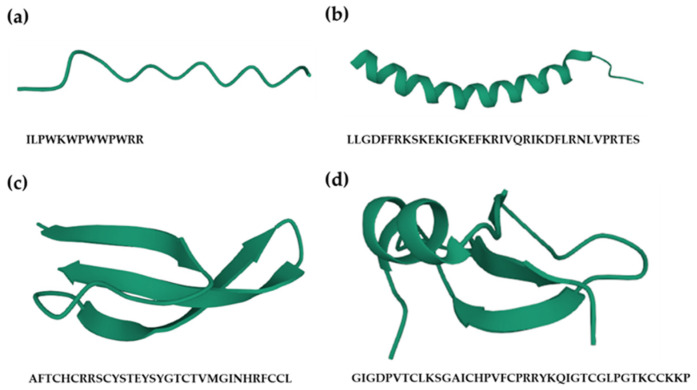
Structures of antimicrobial peptides (AMPs). Based on their structures, AMPs can be divided into four categories, including (**a**) linear peptide, e.g., bovine antimicrobial peptide indolicidin (protein databank, PDB: 1G8C); (**b**) α-helical peptide, e.g., human host defense cathelicidin LL-37 (PDB: 2K6O); (**c**) β-sheeted peptide, e.g., human α-defensin-6 (PDB: 1ZMQ); (**d**) peptide including both α-helix and β-sheet, e.g., human β-defensin-2 (PDB: 1fd3). All the figures were created using an online 3D view (https://www.rcsb.org/3d-view, accessed on 1 December 2021).

Based on their structures, AMPs can be divided into four categories (Figure 1), including linear (bovine indolicidin [65]), α-helix (human cathelicidin LL-37 [66]), β-sheet (human α-defensin 6 [67]), and both α-helix and β-sheet peptides (human β-defensin-2 [68]). The structures of AMPs are changed according to environmental conditions, which is associated with the change of hydrophobicity and net charge of the cell membrane [69].

### 4.2. Killing Mechanisms of AMPs

**Figure 2:**
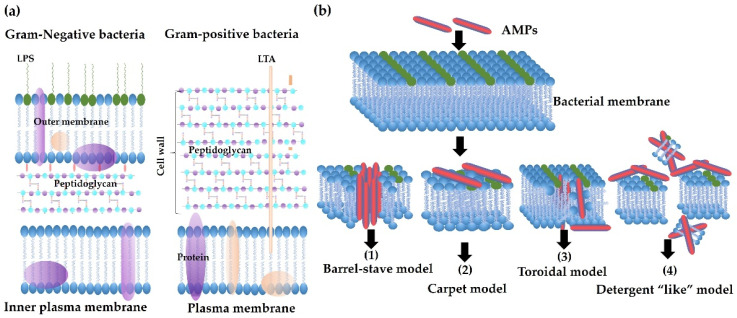
Bacterial membrane structures and mechanisms of action of antimicrobial peptides (AMPs). (**a**) Schematic membrane structures of Gram-positive and Gram-negative bacteria. The cytoplasmic membranes of them are similar. Gram-negative bacteria have a thin layer of peptidoglycan, with lipopolysaccharide (LPS) in the outer membrane. In contrast, Gram-positive bacteria have a thick layer of peptidoglycan surrounding the cytoplasmic membrane, with lipoteichoic acid (LTA) across the peptidoglycan layer. Both LPS and LTA are the binding targets of AMPs. (**b**) Mechanisms of action of AMPs. Membrane-active AMPs interrupt the integrity of the membrane by forming different pores as in the following models: (1) Barrel-Stave model: AMPs perpendicularly insert into the lipid bilayer of the membrane and form a channel. (2) Carpet model: AMPs cover the surface of the membrane without forming specific pores. (3) Toroidal pore model: AMPs also insert perpendicularly in the lipid bilayer without specific peptide–peptide interactions to form a channel. (4) Detergent-like mode: AMPs work like a detergent to break membranes into small pieces.

The net charge and hydrophobicity of AMPs are two important characteristics for the initial binding of AMPs to bacterial membranes. For example, AMPs with a net positive charge can electrostatically interact with negatively charged cell wall components (Figure 2a), such as lipopolysaccharide (LPS) and lipoteichoic acid (LTA), and the interaction of AMPs with LPS can lead to membrane destabilization and permeabilization [70,71]. Then, the hydrophobic residues (e.g., tryptophan) enable AMPs to further insert into the bilayer of the bacterial membrane [72]. The damage of integrity of bacterial membrane results in cell lysis due to a high cytoplasmic osmotic pressure. Release of cytoplasmic contents such as ATP and DNA or RNA can be applied to monitor the bactericidal activity of AMPs and bacterial membrane permeability [70].

Pores formed by membrane-active AMPs can be further divided into four types, including the barrel-stave, carpet, toroidal, and detergent-like models [73,74,75], according to the amino acid residues, hydrophobicity, charge, and length of AMPs. The direct killing mechanism of AMPs is summarized in Figure 2b, which lists four models of action of membrane-active AMPs. In addition, AMPs can penetrate the membrane bilayers and impact the synthesis of DNA, RNA, and proteins.

### 4.3. Immunomodulatory Function of AMPs

Some AMPs have both bactericidal and immunomodulatory functions, such as LL-37 [76], human β-defensin 2 (hBD2) [77], and avian β-defensin-12 [78]. Firstly, AMPs display chemokine-like functions. For example, defensins such as hBD2 and hBD3, as well as their mouse orthologs mBD4 and mBD14, can chemoattract leukocyte migration (e.g., dendritic cells, macrophages, and monotypes) via chemokine receptors CCR6 and CCR2 [77]. Secondly, AMPs can modulate pro-inflammatory function. For example, hBD3 can inhibit Toll-like receptor 4 (TLR4)-mediated pro-inflammatory cytokine expression on activated macrophages in myeloid differentiation factor 88 (MyD88) and Toll/interleukin-1 receptor-domain-containing adapter-inducing interferon-β (TRIF)-dependent signaling pathways [79]. In addition, human β-defensin DEFB126 showed highly binding and neutralizing LPS ability, so it can inhibit LPS-induced inflammatory cytokines such as IL-1β, IL-6, and TNF-α in macrophages [79]. Human cathelicidin LL-37 impacts T cell differentiation, inducing Th17 and suppressing Th1 differentiation during inflammation [80], contributing an important role in autoimmune diseases [20].

### 4.4. Other Functions of AMPs

Some AMPs have a high binding affinity for an anionic membrane to induce membrane depolarization to cause bacterial death. Dye such as 3,3′-Dipropylthiadicarbocyanine iodide or DiSC3(5) can be applied to test the ability of AMPs to depolarize bacterial membranes, which show a low fluorescence emission signal when binding to with polarized membranes of bacteria and increases its fluorescence emission intensity while binding membrane of depolarization [81].

AMPs can also induce cell apoptosis by regulating the production of reactive oxygen species (ROS). For example, psacotheasin, a knottin-type AMP, can trigger apoptosis of *Candida albicans* by inducing the accumulation of ROS [82], specifically hydroxyl radicals. In addition, it also caused depolarization of mitochondrial membrane observed by a cell-permeant, green-fluorescent, lipophilic dye staining.

Cell-penetrating peptides (CPPs) can be developed to transport specific macromolecules intracellularly, including DNA/RNA, plasmids, antibodies, and nanoparticles [83]. CPPs with antimicrobial activity are very effective against intracellular bacterial infection [84,85]. In addition, AMPs can be conjugated with CPPs to improve their ability against intracellular bacterial infection [86], such as *Salmonella* Typhimurium.

## 5. Design and Optimization of AMPs

AMPs show the promising capability to kill MDR-bacteria in vitro, especially when measuring their minimum inhibitory concentrations (MICs) and minimum bactericidal concentrations (MBCs). As above-mentioned mechanisms, bacteria develop resistance mechanisms to abrogate the bactericidal activity of AMPs, such as the formation of biofilms. Modification of AMPs such as conjugating hydroxyapatite to innate defense regulator (IDR)-1018 (VRLIVAVRIWRR) can improve the killing ability against biofilm-producing bacteria [87]. In the following context, the source of AMPs and their design and optimization are discussed.

### 5.1. Natural Peptides

Natural AMPs are found in plants, vertebrates, invertebrates, and small organisms such as bacteria and fungi. Except for animals, there are many different types of AMPs in plants with anti-bacterial, anti-fungal, and insecticidal activities, as well as anti-cancer ability [88,89], such as thionins, defensins, lipid transfer proteins, hevein-like peptides, knottin-type peptides, α-hairpinins, snakins, and cyclotides. Plant AMPs also play important roles in the plant immune system in response to pattern-recognition receptor signaling pathways [90]. In silico strategies can be used to search natural AMPs in the genome, proteome, and transcriptome [91,92,93].

### 5.2. Signaling Peptide-Derived AMPs

Porto et al. reported that a novel cationic AMP can be designed from a signal peptide sequence (i.e., EcDBS1, MKKLFAALALAAVVAPVW) from *E. coli* by Joker algorithm [94]. The modified peptide (EcDBS1R6, PMKKLFKLLARIAVKIPVW) is able to kill bacteria by acting on bacterial membranes [95].

### 5.3. Structural Modification-Hybridization, Shorten, or Circulation

A hybrid peptide, linking a *P. aeruginosa* targeting peptide PA2 (SQRKLAAKLTSK) selected by phage display-assay and an α-helical AMP GNU7 (RLLRPLLQLLKQKLR) with three glycines (-GGG-), displayed selective and strong killing ability against *P. aeruginosa* both in vitro mixed cell culture and in a murine model [96]. Most AMPs are cationic; therefore, they display low bactericidal activity in high salt conditions due to the competent binding activity of cationic ions with bacterial membrane [97]. A chimeric peptide H4 that is derived from hBD3 and hBD4 exhibited stronger antimicrobial activity against bacteria such as *Enterococcus faecalis* and *S. aureus*, with antibacterial activity in high salt conditions [98]. In addition, the N-terminal deletion of three amino acids of hBD3 improved its antimicrobial activity against different bacterial species such as *E. coli* and *Enterococcus faecium*, especially in high salt conditions [99].

Natural θ-defensins in rhesus macaques display antimicrobial activity against bacteria and fungi at low concentrations. For example, θ-defensin-1 (RTD-1) showed 3-fold higher killing activity compared to the open-chain analog, which was not salt-sensitive [100].

### 5.4. In Silico Design

Based on the current antimicrobial peptide database, the AMP motif can be analyzed using a computer and online software to design novel AMPs. For example, two AMP motifs (A15_B and A15_E) were screened by the Support Vector Machines algorithm from Pleurocidin, an AMP found in fish, displayed antimicrobial potentials in silico [101]. Research studies showed that DP7, an AMP designed in silico, showed broad-spectrum antimicrobial activity against MDR bacteria, such as *P. aeruginosa* [102]. Currently, there are many antimicrobial peptides databases (APDs) such as APD3 [63] and collection of antimicrobial peptides (CAMP)R3 [103], as well as online tools for AMP screening and identification such as dbAMP [104] and Ensemble-AMPPred [105].

## 6. Optimization of AMPs

Some AMPs show in vitro capability of killing pathogenic bacteria with promising values of MICs and MBCs. However, the antimicrobial activity of AMPs is compromised in vivo due to high salt concentration, pH change, and enzyme cleavage [106]. Thus, modification or optimization of AMPs to increase their killing efficacy is critically important for their application.

### 6.1. Substitution

Amino acid substitution is a commonly used strategy to improve the killing activity of AMPs, including the substitution of natural L-amino acids with D or unnatural amino acids. For example, peptide UP09 (AibRLFKKLLKYLRKThi, Aib and Thi denote 4-aminobutanoic acid and L-thienylalanine, respectively), derived from cationic AMP Pep05 (KRLFKKLLKYLRKF) by substituting N-terminal and C-terminal amino acids with unnatural amino acids, showed higher antimicrobial activity against *P. aeruginosa* and lower cytotoxicity to host cells in vivo [107].

For cationic AMPs, the charge and hydrophobicity are critically important for their activity. For example, a magainin II analog, P24 (GRAHMRWLRRWRRWGRAWVRILRR) with Lys (K), His (H), Ser (S) residues substituted with Arg (R) and hydrophobic Phe (F) replaced with Trp (W), displayed stronger antimicrobial activity against both Gram-negative (*K. pneumoniae*) and Gram-positive bacteria (*S. aureus*) compared to another magainin II analog pexiganan (GIGKFLKKAKKFGKAFVKILKK) [108].

The online database of antimicrobial activity and structure of peptides (DBAASP) showed that an abundance of bulky hydrophobic and/or aromatic amino acids (Phe, Ile, Leu, Trp, and His) is shown in the feature of linear AMPs, while Cys, Lys, and Gly are rich in cyclic and disulfide-bonded peptides, and Pro, Ser, and Thr are increased in cyclic peptides [109]. In addition, unnatural amino acid residues have been applied in AMPs to improve their killing efficacy and proteolytic resistance, such as 4-aminobutanoic acid and azulenyl-alanine [107,110,111].

### 6.2. N-Terminal Acetylation and C-Terminal Amidation

Cytotoxicity assay showed that N-terminal acetylation and C-terminal amidation of β-hairpin AMP tachyplesin I had higher toxicity to both tumor and normal human cells, with increased hemolytic acidity [112]. However, the modified tachyplesin I was resistant to proteolytic degradation in human serum compared to the original molecule. Another study showed that N-terminal acetylation and C-terminal amidation of CPPs can reduce cell internalization but not alter their toxicity [113].

### 6.3. Fatty Acid Modification

N-terminal myristoylation via conjugating myristic acid to porcine myeloid antimicrobial peptide-36 (PMAP-36) analog PMAP-36PW can improve their permeabilization activity on Gram-negative bacteria and anti-biofilm activity [114]. N-terminal cholesterol-modified peptide PMAP-37(F34-R) improved antibacterial activity against *S. aureus*, displaying anti-biofilm activity and high stability in different pH conditions, as well as resistance to salt, serum, and boiling [115].

### 6.4. Conjugation with Membrane-Binding or Penetrating Peptides

The development of smart chimeric peptides (SCPs) is another strategy to improve the antimicrobial activity of AMPs. For example, a SCP by connecting LPS-binding peptide (LBP)14 with a marine AMP-N6 exhibited increased killing activity against MDR *E. coli* and neutralized LPS ability both in vitro and in vivo [116]. Conjugation of CPPs to AMPs can also enhance their bactericidal activity. For example, conjugation R9 (RRRRRRRRR) with magainin (GIGKWLHSAKKFGKAFVGEIMNS) or M15 (KWKKLLKKLLKLLKK) with three glycines (Gly, G) increased 2 to 4-fold antimicrobial activity against Gram-positive bacteria such as *S. aureus* and *E. faecalis*, and 4 to 16-fold against Gram-negative bacteria such as *E. coli* and *P. aeruginosa* [117].

### 6.5. Modification of AMPs with Organometallic Agents

Organometallic AMPs (OM-AMPs) obtained by conjugating AMPs to organometallic agents (e.g., ferrocene) can significantly increase their killing activity against MRSA. Starting from a hexapeptide (RW)3 structure, modification via changing peptide sequence and position of the organometallic group and by optimizing amino acid chirality significantly improved the antibacterial activity of OM-AMPs [118].

### 6.6. Structural Modification

As discussed above, AMPs display different structures. Modification of AMP structure can also improve their activity and stability. For example, stapling AMPs to a helical structure can increase their resistance to protease by hiding the proteolytic targets [119]. An α-helical structure may also increase the antimicrobial activity of AMPs, such as a melittin-relative peptide (AR-23) [120], and decrease their cytotoxicity such as anti-fungal peptide Cm-p5 [121]. Design of side-chain hybrid dimer AMPs by linking Anoplin (GLLKRIKTLL-NH2) and RW (RRWWRF-NH2) showed a 4 to 16-fold increase of antimicrobial activity compared to parental peptides against *E. coli*, *S. aureus*, *P. aeruginosa*, and *Bacillus subilits* [122].

Furthermore, lipophilicity is a major factor impacting the antimicrobial activity of small cationic lipopeptides [123]. In addition, the lipophilicity and affinity of AMPs or small lipopeptides with antimicrobial activity are related to their killing ability and hemolytic property of peptides [124,125]. AMPs may be disordered in a solution, molecular dynamics simulation can be applied to study the structure of AMPs while they exert their antimicrobial function, such as interacting with lipid membrane [126]. Assays including antimicrobial activity test, hemolytic activity or cytotoxicity, chemotactic activity, inhibition serum inhibition assay, and LPS neutralization assay are commonly applied to evaluate the antimicrobial and chemotactic activity activities of AMPs [127].

**Figure 3:**
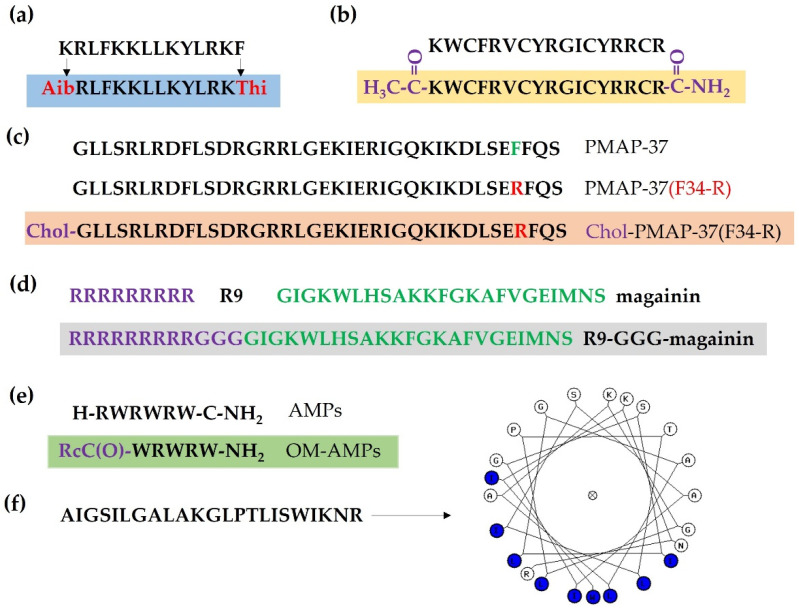
Modification of antimicrobial peptides (AMPs). (**a**) Substitution natural L-amino acids with D- or unnatural amino acids. Aib and Thi denote 4-aminobutanoic acid and L-thienylalanine, respectively. (**b**) N-terminal acetylation and C-terminal amidation of tachyplesin I. (**c**) N-terminal cholesterol-modified peptide PMAP-37 (F34-R). (**d**) Conjugation of R9 (RRRRRRRRR) with magainin (GIGKWLHSAKKFGKAFVGEIMNS) with three glycines (Gly, G). (**e**) Organometallic AMPs (OM-AMPs). (**f**) Design of a helical structure of AMP.

Overall, the goal of modification of AMPs is to enhance their stability and efficacy and to decrease their cytotoxicity and untargeted side effect (Figure 3).

## 7. Delivery

**Figure 4:**
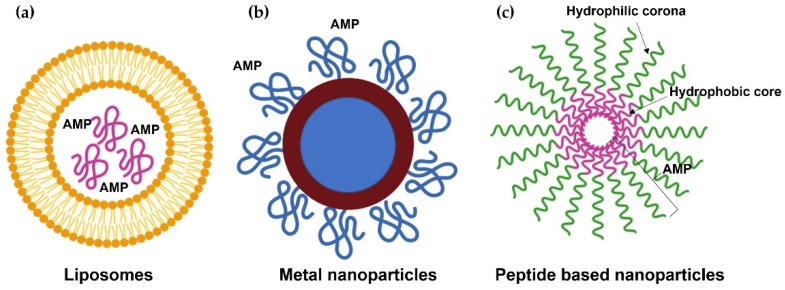
Delivery of antimicrobial peptides (AMPs) by nanoparticles. (**a**) Liposomal system for AMP delivery. (**b**) Metal-based nanoparticles for AMP delivery. (**c**) Self-assembling nanoparticles.

Nanotechnology provides strategies for the delivery of AMPs, promoting their stability, toxicity, and target selectivity [128]. For example, AMPs are sensitive to proteolytic enzymes, which limits their application. Post-exposure to proteolytic enzymes, nano-formed PA-13 that was encapsulated electrostatically into nanoparticles kept their killing activity against *P. aeruginosa* both for in vitro culture and ex vivo skin model in porcine. However, unencapsulated PA-13 lost antimicrobial activity [129]. In addition, nano construction can be applied to design nontoxic AMPs [130]. Here, we discuss some forms of nanoparticles to deliver AMPs (Figure 4).

### 7.1. Lipid-Based Nanoparticles

The liposomal system has been broadly applied to deliver anti-cancer drugs with several favorable characteristics such as physical and chemical stability, reducing cytotoxicity to normal cells [131]. A novel antimicrobial peptide (WLBU2)-modified liposomes showed strong antimicrobial activity against MRSA and *P. aeruginosa* [132]. Using nanoparticles to deliver AMPs can enhance their half-life time and avoid proteolytic degradation [133].

### 7.2. Metal-Based Nanoparticles

Silver nanoparticles (AgNPs) exhibit antimicrobial activity against bacteria both in vitro and in vivo [134,135]. The combination of AgNPs and peptide Tet-213 KRWWKWWRRC) presented a synergistic bactericidal activity [136]. Similarly, gold nanoparticles (AuNPs) show broadly antimicrobial activity against waterborne bacterial pathogens, such as *E. coli*, *S.* Typhimurium, and *Shigella dysenteriae* [137]. AMP-conjugated AuNPs displayed increased antimicrobial activity and stability in serum and low cytotoxicity to human cells [138]. For example, esculentin-1a (an AMP derived from frog skin) coated AuNPs can damage bacterial membrane at low concentration and is more resistant to proteolytic digestion, displaying wound healing ability [139]. In addition, titanium dioxide (TiO_2_) and zinc oxide (ZnO) can be applied to engineer nanomaterials [140].

### 7.3. Self-Assembling Nanoparticles

Self-assembling peptide nanomaterials exhibit several advantageous properties, such as low toxicity and resistance to high salt conditions as well as protease degradation. In addition, they are injectable and biocompatible and are widely applied in drug delivery and nanobiotechnology [141]. C-terminally myristoylation of human α-defensin 5 (HD5) caused formation of a self-assembled nanobiotic, significantly improving the bactericidal activity against *E. coli* and MRSA both in vitro and in vivo. In addition, the self-assembled HD5 displayed minimal hemolytic activity and low toxicity in vivo [142]. Self-assembling peptide dendron nanoparticles, such as C_16_-3RP nanoparticles, display increased bactericidal activity against Gram-negative bacteria with negligible toxicity and show resistance to high salt conditions and protease degradation [143]. Electrostatic or hydrophobic interaction, hydrogen bonding, and π-π stacking interaction between aromatic rings play important roles in peptide self-assembly [144].

## 8. Clinical Application

AMPs have been shown the potential in application against infection of drug-resistant pathogens. Furthermore, many clinical trials are undergoing to evaluate the efficacy of AMPs. Here, some representative clinical trials within the last five years are summarized (Table 3, accessed on 20 December 2021). Several challenges should be overcome to improve the application of AMPs, including high production cost, low bio-stability, and side toxicity [145]. For example, colistin in combination with a carbapenem (e.g., meropenem) shows a synergistic effect against carbapenem-resistant Gram-negative bacteria (e.g., *Acinetobacter baumannii*) in vitro [146,147]. However, a clinical trial (NCT01732250, ClinicalTrials.gov) revealed that there was no significant difference between colistin monotherapy and combination treatment [147]. Using the above-discussed strategies, AMPs can be modified or optimized to improve their bioactivity and stability and reduce the production cost (e.g., truncated or short AMPs) and cytotoxicity. Overall, more clinical trials are required to further validate the antimicrobial and immunomodulatory functions of AMPs.

Although there is less of a tendency for bacteria to develop resistance to AMPs compared to antibiotics, resistance to AMPs cannot be ignored [158,159]. In addition, currently, it is still unrealistic to completely abandon the use of antibiotics. Therefore, combinatory treatment of AMPs with conventional antibiotics provides a strategy to improve bactericidal activity and reduce antibiotic resistance [160,161]. For example, cyclic peptide [R4W4] in combination with antibiotic tetracycline significantly increased bactericidal activity against multidrug-resistant MRSA and *E. coli* compared to tetracycline alone [85].

## 9. Conclusions

Overuse and misuse of antibiotics accelerate the development of antibiotic resistance. AMPs with broad-spectrum antimicrobial activity and immunomodulatory function are promising antibiotic alternatives. The dual mechanisms of function of AMPs make bacteria develop hardly any resistance to AMPs. However, the application of AMPs is impacted by several barriers including their stability, salt sensitivity, hemolytic activity, and unpredicted toxicity, which causes the current use of AMPs mainly applied to topical infections. AMP-based nanomedicines can be designed to avoid the above barriers of AMP application. Research studies focusing on improving the antimicrobial activity of AMPs in vivo and targeted delivery are still the objectives in the following decade since AMPs are potential agents against MDR bacterial infections. More clinical trials are waiting to be investigated for the application of AMPs or agents that can modulate endogenous AMP expression. In addition, combinatory treatment of AMPs with conventional antibiotics can improve bactericidal activity and reduce antibiotic resistance.

## Figures and Tables

**Table 1:** The killing mechanisms of antibiotics and resistance mechanisms of bacteria.

Antibiotics/Classes	Mode of Action	Bacteria	Mechanism of Resistance	References
Penicillin and carbapenem (beta-lactam)	Inhibiting bacterial cell wall synthesis	*Escherichia coli* and *Klebsiella pneumoniae*	Producing beta-lactamase and carbapenemase and porin alteration	[26]
Macrolides	Inhibiting protein synthesis by binding to the 50S ribosomal subunit	*K. pneumoniae*	Producing erythromycin esterases (Eres) such as EreA and EreC	[27,34]
Ticarcillin (beta-lactam) and ciprofloxacin (quinolone)	Inhibiting bacterial cell wall and protein synthesis	*Pseudomonas aeruginosa*	Resistance-nodulation-division (RND) efflux pumps	[29]
Macrolides	Inhibiting protein synthesis	*Streptococcus pneumoniae*	Ribosomal demethylation, expelling by efflux pump, and target site mutation	[30]
Quinolones	Inhibiting nucleic acid synthesis	*K. pneumoniae* and *Clostridium perfringens*	Mutations in the genes that encode gyrase and topoisomerase IV	[31,32]
Trimethoprim-sulfamethoxazole	Inhibiting folate synthesis	*Burkholderia pseudomallei*	Structural modification of dihydrofolate reductase (DHFR) or dihydropteroic acid synthase (DHPS)	[33]

**Table 2:** Mobile genetic elements in bacteria responsible for antibiotic resistance.

Bacterial Strains	Mobile Genetic Elements	Resistance to Antibiotics	References
*E. coli*, *K. pneumoniae*, and *A. baumannii*	Plasmid-encoded tigecycline resistance *tet(X3)* and *tet(X4)* genes.	Tigecycline	[42,43]
*Pseudomonas* spp.	Plasmid-mediated quinolone resistance (qnr) genes such as *qnrD*, *qnrS*, and *aac(6’)-Ib-cr.*	Quinolone	[37]
Gram-negative bacteria such as *E. coli and* *P. aeruginosa*	Plasmid-mediated AmpC β-lactamases genes *bla*_CITM_ and *bla*_DHAM_ genes	Beta-lactam antibiotics such as ceftazidime, cefepime, and cefoxitin	[38]
*Enterobacterales*	Tn7-like transposons such as Tn6813, Tn6814, and Tn6765.	Sulfamethoxazole and streptomycin	[45]
*Acidaminococcus intestine*	Beta-lactamase encoded gene *aci-1* is found in transposons of in human microbiota, which causes resistance to β-lactam antibiotics.	Beta-lactam antibiotics such as penicillin	[39]
*E. coli*	Class 1 integrons associated with tetracycline-resistant genes *tet(A)* and *tet(B).*	Tetracycline	[40]
Bacteria such as *E. coli* and *Enterobacteriaceae*	Bacteriophage-carried resistance genes such as *bla_TEM_*, *qnrA*, *mecA*, and *sul1*.	Penicillin, quinolone, methicillin, sulfonamide	[50,53]
*E. coli*	ARGs were found in agricultural soil and fresh vegetables such as lettuce and cucumber, including *bla_TEM_* and *qnrA*.	Penicillin and quinolone	[54]
*A. baumannii*	Phage-carried antimicrobial resistance genes carbapenemase gene *OXA-23* and New Delhi metallobeta-lactamase 1 (*NDM-1*).	Beta-lactam antibiotics such as carbapenem	[55]
*E. coli*	ESBL-encoding genes (e.g., *bla*_CTX-M-15_) in *E. coli* include at least three types of mobile elements including plasmids, bacteriophages, and transposon.	Beta-lactam antibiotics such as carbapenem	[56]

**Table 3:** Completed clinical trials with studies relative to AMPs.

Trial Number	Phase	Results	Reference
NCT01959113	1	AMPs secreted by commensal coagulase-negative *Staphylococcus* in healthy skin displayed selectively antimicrobial activity against *S. aureus*.	[148]
NCT01967628	1	Supplementation of vitamin D_3_ during increased AMP (e.g., LL-37) concentration in airway surface liquid in the Winter and Spring seasons.	[149]
NCT01372995	2	Treatment with a high-dose vitamin D_3_ can increase the expression of human cationic antimicrobial protein (hCAP18) mRNA in plasma.	[150]
NCT01447017 NCT01522391	2	DPK 060, an antimicrobial peptide derived from the endogenous protein kininogen, was an effective and safe drug candidate for the topical treatment of microbial infections.	[151]
NCT02456480	2	Treatment with topical omiganan, an indolicidin analog, significantly improved the local objective scoring atopic dermatitis index in patients.	[152]
ISRCTN12149720	2	Treatment of anti-biofilm peptide P60.4Ac-containing ototopical drops was safe and well-tolerated, with 47% of successful cases for patients suffering from chronic suppurative otitis media.	[153]
IRCT20090822002365N17	3	Supplementation of CoQ10 dramatically increased serum levels of cathelicidin LL-37.	[154]
ChiCTR-OIC-16010250	3	Nal-P-113, an AMP P-113 with histidine residues replaced by β-naphthylalanine, can restrain the growth of *Streptococcus gordonii*, *Fusobacterium nucleatum*, and *Porphyromonas gingivalis* and biofilm formation at a concentration of 20 μg/mL.	[155]
NCT00310726	None	Polymorphisms in the human β-defensin 1 gene were negatively and significantly associated with HIV-1 infection in the Zambian population.	[156]
NCT03622918	None	The colistin/rifampicin combination treatment induced a higher microbiological response rate in patients with pneumonia induced by colistin-resistant *Acinetobacter baumannii*.	[157]

## Data Availability

All data supporting reported literature can be found in this paper.
